# Structure Reports Online: successful transition to open access

**DOI:** 10.1107/S1600536808042979

**Published:** 2009-01-10

**Authors:** William T. A. Harrison, Jim Simpson, Matthias Weil

**Affiliations:** a Department of Chemistry, University of Aberdeen, Aberdeen AB24 3UE, Scotland; b Department of Chemistry, University of Otago, PO Box 56, Dunedin, New Zealand; c Institute of Chemical Technologies and Analytics, Division of Structural Chemistry, Vienna University of Technology, Getreidemarkt 9/164-SC, Austria

In the editorial published this time last year, the founding Section Editors of the journal, Bill Clegg and David Watson, signalled their intention to retire in August at the IUCr Congress in Osaka, Japan. This has now taken place and the three of us, Bill Harrison from Scotland, Jim Simpson from New Zealand and Matthias Weil from Austria, have taken on the daunting task of replacing them. What better way to start this editorial than to acknowledge the fantastic contribution of the retiring *Acta E* Section Editors? Bill and David brought this journal from a concept to an extremely successful reality and more recently oversaw the change to the new short-format papers and steered *Acta E* through the potentially turbulent process of becoming open access, ensuring throughout that the papers published in the journal maintained a high degree of quality and consistency. It is our task now to keep up the extremely high standards that they achieved. Thank you both for all you have done for *Acta E* and enjoy your ‘retirement’, at least from the Section Editors’ jobs.

At the recent Commission on Journals meeting, held in association with the Osaka Congress, it was obvious that the move to open access for *Acta E* has not only been successful, but has also allayed the fears of many sceptics who felt that the requirement to pay for publication would lead to the journal’s demise. The present funding model appears secure and there are no plans to increase the open-access charge for the journal in 2009. The continued growth of *Acta E* in the post open-access period was strongly welcomed by members of the Executive Committee and we, as Section Editors, will do all that we can to maintain and build on this successful outcome.

Following a decline in submissions in the first three months after the journal became open access, the number of papers submitted each month has increased gradually to a level approaching that of 2006. Table 1 ▶ compares monthly submissions for the past four years. Clearly the deluge of papers that flooded into the submission system in October and November of 2007 is unlikely to be repeated. Nonetheless, the steady increase in submissions throughout 2008 augurs well for the future of the journal.

The total number of papers published in 2008 was 3556, compared with 5181 in 2007, 3991 in 2006 and 2887 in 2005. Of these 63% described structures of organic, 34% structures of metal-organic and 3% structures of inorganic compounds. 51% of authors were from China, 6% from India, 5% from the USA, 4% from Germany, 4% from Malaysia, 3% from Iran and 2% from Pakistan with smaller percentages from other countries. The number of papers withdrawn or rejected for a variety of reasons remains similar at 16%, while the journal’s official impact factor is 0.508.

The *checkCIF* software continues to be developed thanks to the efforts of Ton Spek and Mike Hoyland. Recently, some new checks on submitted structure factor (.fcf) files have been incorporated into the submission process. Authors should check for any additional alerts once all of the files for their paper have been submitted. These additional checks effectively compare values reported in the CIF with the contents of the structure-factor file and other metrical information that can be calculated directly from the reflection data. Missing reflections out to θ_max_ and reflections that may be affected by the beamstop are also indicated.

As new Section Editors we are delighted to have inherited a cohort of more than 50 Co-editors whose knowledge and expertise is of enormous benefit to us, the journal and the small molecule crystallographic community. Dr J. Low, Professor A. Roodt, Professor A. M. Z. Slawin and Professor B. M. Yamin retired as Co-editors during last year and we are very grateful to them for their work on behalf of the journal. We expect that there will be a small number of additions to the Co-editorial board in 2009 to accommodate the steady increase in submissions and to spread the load among the hard working Co-editors. These new appointments will be announced in a future editorial. Our task is also made so much easier by the excellent work of the IUCr editorial staff in Chester, to whom we express our heartfelt thanks. Without their input, the journal would simply not function.

## Figures and Tables

**Table 1:** Papers submitted to *Acta E* in the period 2005–2008

Year	Jan	Feb	Mar	Apr	May	Jun	Jul	Aug	Sep	Oct	Nov	Dec	
2005	318	185	309	310	279	320	291	266	377	401	376	247	
2006	330	293	379	346	419	416	463	475	507	539	562	378	
2007	458	350	681	445	521	518	487	465	450	785	1066	228	
2008	231	155	286	352	298	326	377	282	383	392	417	347	

